# Occupational Stress and Mental Health: A Comparison Between Frontline Medical Staff and Non-frontline Medical Staff During the 2019 Novel Coronavirus Disease Outbreak

**DOI:** 10.3389/fpsyt.2020.555703

**Published:** 2020-12-23

**Authors:** Xie Zhang, Ke Zhao, Guohua Zhang, Ruihua Feng, Jianjun Chen, Dongwu Xu, Xiaodong Liu, Arlette J. Ngoubene-Atioky, Hong Huang, Yanlong Liu, Li Chen, Wei Wang

**Affiliations:** ^1^Department of Pharmacy, Ningbo Medical Center Li Huili Hospital, Ningbo, China; ^2^The Affiliated Kangning Hospital, Wenzhou Medical University, Wenzhou, China; ^3^School of Mental Health, Wenzhou Medical University, Wenzhou, China; ^4^Center for Health Assessment, Wenzhou Medical University, Wenzhou, China; ^5^Center for Psychology, Goucher College, Baltimore, MD, United States

**Keywords:** occupational stress, COVID-19, medical staff, depression, anxiety, insomnia

## Abstract

**Background:** During an epidemic, both frontline and non-frontline medical staff endure stressful work circumstances that render their mental health a major public health concern. This study aims at investigating and comparing the prevalence and severity of mental health symptoms (i.e., anxiety, depression and insomnia) between frontline medical staff and non-frontline medical staff during the coronavirus disease 2019 (COVID-19) outbreak. It also seeks to evaluate the association of their mental health with occupational stress.

**Methods:** A cross-sectional study was conducted in Wenzhou, China from 2020 February 16th to 2020 March 2th. A total of 524 medical staff responded to the Generalized Anxiety Disorder Scale, the Patient Health Questionnaire, the Insomnia Severity Index, the Occupational stress Questionnaire, and a demographic data form. Data were principally analyzed with logistic regression.

**Results:** Of the 524 participants, 31.3% reported depression, 41.2% reported anxiety, and 39.3% reported insomnia. Compared with the citizens during the COVID-19 epidemic, medical staff experienced higher level of anxiety, depression and insomnia, especially the frontline medical staff. Furthermore, male, married medical staff with poorer physical health reported lower mental health. Frontline medical staff endorsed higher self-reported occupational stress, especially higher occupational hazards, than non-frontline medical staff. In addition, four indicators on occupational stress (working intensity, working time, working difficulty and working risk) were correlated positively with mental health symptoms. Regression analyses found a significant association between occupational stress and mental health symptoms in both frontline and non-frontline medical staff during COVID-19 outbreak.

**Conclusion:** The results indicated that during the COVID-19 epidemic, medical staff experienced higher levels of anxiety, depression and insomnia than citizens, and their occupational stress had positive effects on their psychological distress. These findings emphasize the importance of occupational stress management interventions to decrease the risk of developing mental health problems among the medical staff during a biological disaster.

## Introduction

The World Health Organization has declared the coronavirus disease 2019 (COVID-19) outbreak a worldwide pandemic. By the end of April 2020, COVID-19 has spread in more than 140 countries and has infected more than two million people. An infectious disease outbreak, such as Middle East respiratory syndrome (MERS) and severe acute respiratory syndrome (SARS), is a biological disaster that causes profound fear, anxiety, and panic in individuals subjected to the real or perceived threat of the virus (1, 2). Compared to previous epidemics, COVID-19 is capable of human-to-human transmission, asymptomatic carrier transmission and high transmission efficiency, which makes it challenging and highly stressful for medical staff to treat. Such an occupational environment is likely to impede frontline workers' mental health (3).

Medical practice is known to be stressful (4). Medical staff members, including physicians and nurses, usually experience heavy workloads, extended working hours and high levels of time pressure in routine work (4, 5). Epidemic outbreak could exacerbate occupational stress and even burnout in the medical staff. For example, David Koh and his colleagues found that more than half of clinical staff reported increased work stress (56%) during the SARS epidemic in Singapore (6). In addition, medical workers, especially nurses, were vulnerable to many occupational risks and experienced a great deal of emotional stress related to their work in MERS outbreak (7). During the COVID-19 outbreak, medical staff are inevitably exposed to an extremely stressful work environment with the ever-increasing number of confirmed and suspected patients, overwhelming workload, depletion of personal protection equipment, and severe shortage of manpower (8, 9). Numerous studies indicate that acute and chronic stressful occupational experiences significantly contribute to mental health concerns (10, 11). These studies not only have significantly advanced current knowledge concerning the mental health of frontline medical staff but also have motivated new important research questions. For example, what is the mental health profile of essential medical staff during the COVID-19 outbreak? Do medical staff who are under severe or constant occupational stress during the COVID-19 outbreak experience more mental health problems? Compared with non-frontline medical staff, is it possible for frontline medical staff who are directly involved in the diagnosis, treatment, and care of patients with COVID-19 to be at higher risk of developing psychological distress due to higher occupational hazards and greater work burden?

Therefore, the present study seeks to expand existing studies by (1) investigating and comparing the prevalence of and severity of mental health symptoms between frontline medical staff and non-frontline medical staff during the COVID-19 outbreak, (2) identifying the characteristics of medical staff with the mental health symptoms, and (3) evaluating the association of their mental health with occupational stress.

## Methods

### Participants and Procedure

#### Medical Staff

Due to the COVID-19 epidemic, face-to-face investigations were restricted (12). Therefore, online questionnaire was constructed via a WeChat applet. Data were collected from both the non-frontline medical staff and frontline medical staff during the COVID-19 pandemic from February 16 to March 2, 2020. The online questionnaire for medical staff (doctors, nurses, and medical technician) was publicized through posters in one isolation hospital designated for COVID-19 patients (The First Affiliated Hospital of Wenzhou Medical University) and two common hospitals (Yuying Children's Hospital of Wenzhou Medical University and Wenzhou People's Hospital) in Wenzhou, which is one of the most affected cities in terms of the number of COVID-19 cases apart from those in the hardest-hit Hubei Province in China (13). The participants included frontline medical staff and non-frontline medical staff. Frontline medical staff were defined as the medical workers who directly participated in the fight against COVID-19 by contacting confirmed COVID-19 cases or their specimens in the isolation hospitals. Non-frontline medical staff were defined as the medical workers who deal with non-COVID-19 patients in the common hospitals. All the participants were recruited through purposive sampling by means of Wen Juan Xing (www.wjx.cn), which is a widely used web-based survey platform in China. Participants were assured of data confidentiality and it was explained that only the authorized researchers could access the data. This study was conducted in compliance with the Helsinki Declaration, and was reviewed and approved by the Ethics Committee of Wenzhou Medical University. Hence, 536 medical staff were eligible for the study and consent with the study procedures, and then 524 made valid replies, yielding a response rate of 97.76%.

#### Citizens

In order to compare the level of mental health between the medical staff and citizens, we used the data of mental health of citizens in one study conducted by Mu (14) (“Knowledge, and attitudes toward COVID-19 among Chinese citizens and their mental health during the period of the COVID-19 outbreak”). In this study, a cross-sectional study was conducted by the online questionnaire constructed via a WeChat applet from 10th February 2020 to 20th February 2020 in China. The online questionnaire for citizens was publicized through posters by the community staff in three communities in Beijing, and all subjects voluntarily participated and signed informed consent in this survey and identified by the method of random number. Hence, 217 Chinese citizens were eligible for the study and consent with the study procedures. The prevalence of anxiety and depression was estimated by the Generalized Anxiety Disorder Scale and the Patient Health Questionnaire.

### Measurements

#### Demographic Data

A demographic questionnaire elicited basic background information, including gender, age, education level, marital status, health status, and length of service.

#### Occupational Stress

For the purpose of this study, occupational stress is defined as the stressful aspects of work that a medical staff experienced in their workplace. Four items assessed medical staff occupational stress during the COVID-19 outbreak: (1) work hours, (2) work intensity, (3) work difficulty, and (4) occupational hazards during the COVID-19 epidemic. Responses were recorded on a 5-point scale ranging from “Strongly Disagree” to” Strongly Agree.” Example items include “I have very long working hours during the epidemic” and “I have too much work allotted to me during the epidemic.” Higher scores indicate a higher degree of occupational stress. The items demonstrated acceptable internal consistency in this sample (α = 0.74).

#### Self-Reported Symptoms of Mental Health

##### Anxiety

The Generalized Anxiety Disorder Scale-7 (GAD-7) was used to determine the level of anxiety of participants. The seven items of the GAD-7 measure the frequency by which participants experience within the last 2 weeks the seven core symptoms of GAD (15). Items are rated from 0 (not at all) to 3 (almost every day), such that the total score ranges from 0 to 21. The score is interpreted as indicating either no anxiety (0–4), mild (5–9), moderate (10–14), or severe anxiety (15–21). Previous studies have shown that the GAD-7 is a well-validated screening instrument (16), and it has demonstrated excellent internal consistency (Cronbach's alpha = 0.94) in the present study.

##### Depression

The Patient Health Questionnaire-9 (PHQ-9) (17) is a nine-item assessment tool designed to measure depression based on the nine diagnostic criteria for major depressive disorder covered in the Diagnostic and Statistical Manual of Mental Disorders, 5th edition (DSM-V). Items are rated from 0 (not at all) to 3 (almost every day) according to increased frequency of experiencing difficulties in each area covered within the last 2 weeks. Total score ranges from 0 to 27 and indicates either no depression (0–4), mild (5–9), moderate (10–14), moderately severe (15–19), or severe depression (20–27). The PHQ-9 is a well-validated screening instrument (18) that has yielded strong internal consistency (Cronbach's alpha = 0.87) in the present study.

##### Insomnia

The Insomnia Severity Index (ISI) (19) consists of seven items which corresponds in part to DSM-IV criteria for insomnia. Items are rated from 1 (not at all) to 5 (almost every day), higher scores indicate more severe insomnia. Scores are summed and can range from 0 to 28. The total score signifies either absence of insomnia (0–7), mild (8–14), moderate (15–21), or severe insomnia (22–28). Previous studies have shown that the ISI is a well-validated screening instrument (20), and it has demonstrated excellent internal consistency (Cronbach's alpha = 0.94) in the present study.

### Statistical Analysis

Categorical variables were presented as numbers (percentages) and analyzed using chi squared test. Continuous variables with normal distribution were expressed as mean ± standard deviation and analyzed using independent samples *t*-test, while those with skewed distribution were analyzed using MannWhitney *U*-test. Hierarchical multiple regression models were established to identify factors that contributed to mental health symptoms (i.e., anxiety, depression, and insomnia) in frontline medical staff or non-frontline medical staff. All statistical analyses were performed with SPSS statistics package (version 18.0) and all reported *P*-values are two-tailed with statistical significance set at 0.05.

## Results

### Demographic Characteristics in Frontline Medical Staff and Non-frontline Medical Staff

A total of 524 medical staff from hospitals in Wenzhou completed this survey. Of these participants, 150 (28.6%) are frontline medical staff in direct contact with confirmed COVID-19 patients, and 374 (71.4%) are non-frontline medical staff in direct contact with non-COVID-19 patients. Participants' demographics are shown in Table 1. It is noted that younger (33.65 ± 6.71), more educated (college or above) (96.7%) or unmarried (32%) medical staff were found in frontline medical staff compared with non-frontline medical staff correspondingly (36.10 ± 7.11; 78.9% or 15.2%).

**Table 1:** Demographic characteristics of the respondents (*N* = 524).

**Variables**	**Frontline medical staff (*n* = 150)**	**Non-frontline medical staff (*n* = 374)**	**Statistics**	***p***
Gender			14.79	<0.001
Male (*n* = 134, 25.6%)	21 (14.0%)	113 (30.2%)		
Female (*n* = 390, 74.4%)	129 (86.0%)	261 (69.8%)		
Age (mean ± SD)	33.63 ± 6.72	36.10 ± 7.11	3.02	<0.001
Education level			25.17	<0.001
High school or below (84, 16.0%)	5 (3.3%)	79 (21.1%)		
College or above (440, 84.0%)	145 (96.7%)	295 (78.9%)		
Professional			19.78	<0.001
Nurse (292, 55.7%)	120 (80%)	172 (45.9%)		
Doctor (196, 37.2%)	22 (14.7%)	174 (46.5%)		
Medical technician (36, 7.1%)	8 (5.3%)	28 (7.6%)		
Marital status			18.79	<0.001
Unmarried (105, 20.0%)	48 (32.0%)	57 (15.2%)		
Married (419, 80.0%)	102 (68.0%)	317 (84.8%)		
Health status			2.91	0.08
Good (429, 81.9%)	116 (77.3%)	313 (83.7%)		
Fair or poor (95, 18.1%)	34 (22.7%)	61 (16.3%)		
Length of service (mean ± SD)	10.67 ± 7.49	13.19 ± 9.05	3.65	0.003

*Chi-square analysis was used to test for differences in the categorical variables, and t-test was used to test for differences in the continuous variables between frontline medical staff and non-frontline medical staff*.

### Self-Reported Symptoms of Mental Health in Frontline Medical Staff and Non-frontline Medical Staff

Out of 524 participants, 164 (31.3%) subjects endorsed symptoms of depression on the PHQ-9, 216 (41.2%) subjects reported symptoms of anxiety on the GAD-7, and 206 (39.3%) subjects had symptoms of insomnia on the ISI. Prevalence of insomnia, anxiety and depression was also higher in frontline staff than in non-frontline workers, as shown in Table 2.

**Table 2:** Comparison of proportion of different levels of insomnia, anxiety and depression between non-frontline medical staff and frontline medical staff.

		**Non-frontline medical staff**	**Frontline medical staff**	***χ^2^***	***p***
Depression	Symptom absent	280 (74.9%)	80 (53.3%)	26.78	<0.001
	Mild symptom	79 (21.1%)	51 (34.0%)		
	Moderate symptom	14 (3.7%)	18 (12.0%)		
	Severe symptom	1 (0.3%)	1 (0.7%)		
Anxiety	Symptom absent	220 (58.8%)	88 (58.7%)	9.89	0.02
	Mild symptom	122 (32.6%)	37 (24.7%)		
	Moderate symptom	22 (5.9%)	14 (9.3%)		
	Severe symptom	10 (2.7%)	11 (7.3%)		
Insomnia	Symptom absent	245 (65.5%)	73 (48.7%)	32.68	<0.001
	Mild symptom	105 (28.1%)	48 (32.0%)		
	Moderate symptom	24 (6.4%)	21 (14.0%)		
	Severe symptom	0 (0.0%)	8 (5.3%)		

Moreover, scores on insomnia, anxiety and depression in all participants were 6.74 ± 5.64 in ISI, 4.50 ± 4.40 in GAD-7 and 3.55 ± 3.89 in PHQ-9 scales respectively, which were above the cutoff score for mental health concern in each questionnaire. Medical staff (frontline or non-frontline) scored higher on anxiety (frontline, non-frontline, mean ± standard deviation; GAD-7: 5.10 ± 5.09, 4.26 ± 4.08) and depression (PHQ-9: 4.99 ± 4.45, 2.98 ± 3.49) than citizens (14) during the COVID-19 epidemic (GAD-7: 1.15 ± 2.13, *p* < 0.001, PHQ-9: 0.70 ± 1.89, *ps* < 0.001). Furthermore, compared with non-frontline peers, frontline staff scored significantly higher on insomnia (non-frontline, frontline, mean ± standard deviation; ISI: 5.86 ± 5.05, 8.95 ± 6.39, *p* < 0.001), anxiety (GAD-7: 4.26 ± 4.08, 5.10 ± 5.09, *p* < 0.05) and depression (PHQ-9: 2.98 ± 3.49, 4.99 ± 4.45, *p* < 0.05).

**Figure 1:**
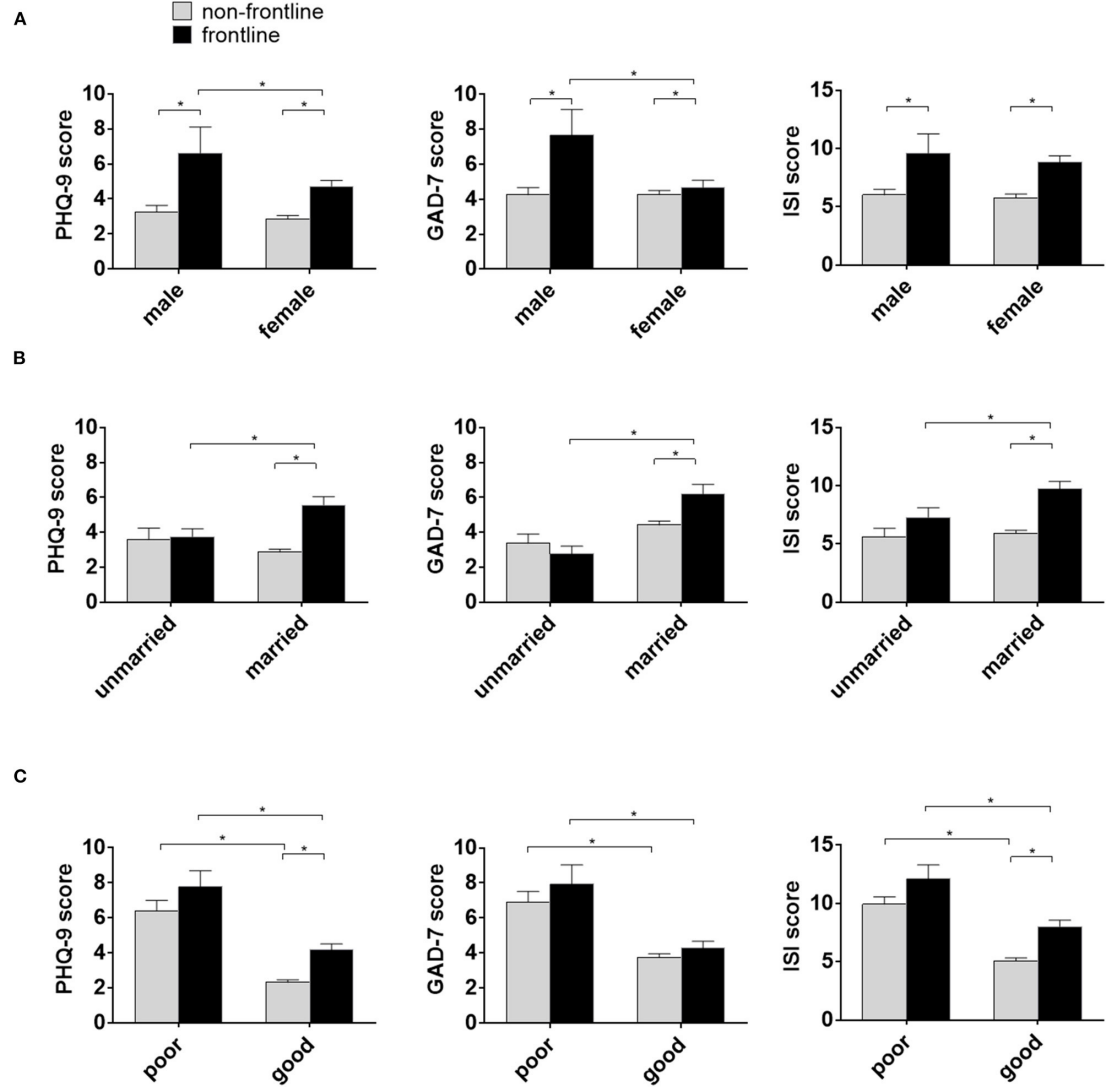
The characteristics of medical staff susceptible for mental health problems during the COVID-19. **(A)** Self-reported depression and anxiety scores higher in male frontline staff compared with female frontline staff. Scores reported in mean ± standard error of the mean(SEM). * =*p* < 0.01. PHQ-0 = Patient Health Questionnaire; GAPD-7 = Generalized Anxiety Disorder 7-item; ISI=Insomnia Severity Index. **(B)** Self-reported depression and anxiety scores higher in married frontline staff compared with unmarried frontline staff. **(C)** Self-reported depression and anxiety scores higher in poor physical health medical staff compared with good physical health staff.

In order to recognize the characteristics of frontline staff susceptible to these mental health problems, multiple comparisons were performed. It was found that among the frontline medical workers, male staff members scored higher on GAD-7 and on PHQ-9. However, this difference was not discovered between male and female non-frontline staff (Figure 1A). In addition, compared with unmarried workers, married medical staff showed similar pattern on insomnia, anxiety and depression scales (Figure 1B). Also, poor physical health had a strong impact on mental health as reflected by the ISI, GAD-7 or PHQ-9 scores, regardless of non-frontline or frontline responsibilities (Figure 1C).

### Characteristics of Occupational Stress in Frontline Medical Staff and Non-frontline Medical Staff

As mentioned above, several characteristics of medical staff had effects on the occurrence of mental health problems during the COVID-19 epidemics, however, the influence of occupational stress on mental health should also be concerned. Occupational stress was characteristic with four indicators including working intensity, working time, working difficulty, and working risk. The mean score of occupational stress in the medical staff was 14.52 ± 2.06. As showed in Table 3, compared with non-frontline staff, frontline staff scored significantly higher in work difficulty and in occupational hazards.

**Table 3:** Comparison of occupational stress between non-frontline medical staff and frontline medical staff.

**Variables**	**Frontline medical staff (*n* = 150)**	**Non-frontline medical staff (*n* = 374)**	**Mann-Whitney U**	***p***
**Occupational stress**				
Working intensity	3.56 ± 0.76	3.53 ± 0.68	28367.00	0.824
Working hours	3.33 ± 0.92	3.59 ± 0.63	23318.00	0.001
Working difficulty	3.67 ± 0.73	3.37 ± 0.62	34445.00	<0.001
Working risk	4.27 ± 0.62	3.90 ± 0.58	36377.00	<0.001

### Hierarchal Multiple Regression Analysis of Factors Contribution to Mental Health Problems in Frontline or Non-frontline Medical Staff

Results from frontline staff multiple linear regression analysis were summarized in Table 4. The ISI (β = −3.69, *p* = 0.002), GAD-7 (β = −3.61, *p* < 0.001) and PHQ-9 (β = −3.90, *p* < 0.001) scores were inversely and strongly associated with physical health status. With regard to occupational stress, only ISI scores were correlated with working risk (β = 2.16, *p* < 0.001). Both GAD-7 (β = 1.35, *p* < 0.001) and PHQ-9 (β = 1.11, *p* < 0.001) scores were related to work difficulty.

**Table 4:** Hierarchical multiple regression analysis of insomnia, depression and anxiety in frontline medical staff.

	**Model**	**Variable**	**B**	**SE**	***P***	***ΔModel R^**2**^***	***P***
Insomnia	1	Health status	−3.69	1.19	0.002	0.07	0.001
	2	Working risk	2.16	0.81	0.008	0.11	<0.001
Anxiety	1	Marital status	2.60	0.80	0.004	0.09	<0.001
	2	Health status	−3.61	0.87	<0.001	0.16	<0.001
	3	Gender	3.46	1.05	0.003	0.21	<0.001
	4	Working difficulty	1.35	0.51	0.008	0.24	<0.001
Depression	1	Health status	−3.90	0.79	<0.001	0.11	<0.001
	2	Education level	3.75	1.84	0.043	0.14	<0.001
	3	Gender	2.32	0.95	0.016	0.16	<0.001
	4	Working difficulty	1.11	0.45	0.014	0.19	<0.001

The same regression model was performed on the non-frontline group with results summarized in Table 5. Physical health status was significantly correlated with GAD-7 (β = −3.22, *p* < 0.001), ISI (β = −4.97, *p* < 0.001) and PHQ-9 (β = −4.01, *p* < 0.001) scores. Interestingly, the regression analysis showed that work difficulty was also related to GAD-7 (β = 1.25, *p* < 0.001), which is consistent with the analysis result from frontline staff. In addition, working intensity was related to insomnia in ISI scores (β = 0.72, *p* < 0.05), and working hour was related to depression in PHQ-9 scores (β = 0.70, *p* < 0.01).

**Table 5:** Hierarchical multiple regression analysis of insomnia, depression and anxiety in non-frontline medical staff.

	**Model**	**Variable**	**B**	**SE**	***P***	***ΔModel R^**2**^***	***P***
Insomnia	1	Health status	−4.97	0.65	<0.001	0.13	<0.001
	2	Education level	1.67	0.59	0.006	0.14	<0.001
	3	Working intensity	−0.72	0.35	0.039	0.15	<0.001
Anxiety	1	Health status	−3.22	0.53	<0.001	0.08	<0.001
	2	Working difficulty	1.25	0.33	<0.001	0.14	<0.001
Depression	1	Health status	−4.01	0.43	<0.001	0.18	<0.001
	2	Working hours	0.70	0.25	0.006	0.19	<0.001

## Discussion

Extending previous research on mental health among medical staff in China, this study investigated and compared the prevalence and severity of mental health symptoms between frontline medical staff and non-frontline medical staff. It also examined whether mental health is associated with four indicators of occupational stress among medical staff in the region with high prevalence of COVID-19 epidemic in China.

To our knowledge, this is the first study to compare the level of mental health between frontline medical staff and non-frontline medical staff during the COVID-19 outbreak. The first finding of the study is that medical staff exhibited much poorer mental health than citizens during the COVID-19 epidemic. Similarly, compared with the non-frontline staff, the frontline medical staff, who had direct and frequent contact with COVID-19 patients, suffered higher level of anxiety, depression, and insomnia. This is consistent with previous reports during severe epidemics outbreak (21–23). For instance, in one study conducted in the SARS outbreak, health care professionals showed higher levels of emotional distress than that of the general public (22). Another study reported that the medical staff in the hospital for SARS infected patients felt extreme vulnerability, uncertainty and threat to life; they also exhibited significantly high psychiatric morbidity of acute stress syndrome (21). The high level of contagion, the unfamiliarity with the characteristics of the virus, the elevated transmission rate, and the experience of isolation increase the psychological burden of medical staff and subsequently, their propensity for mental health problems during COVID-19 outbreak. The present study, along with prior studies, indicates that mental health problem is common among medical staff, especially frontline medical staff.

The second important finding of the study is that male, married medical staff with poorer physical health exhibited much poorer mental health. Firstly, poorer physical health showed strong association with worse mental health of medical staff, no matter whether working in non-frontline or frontline. Literature suggests that excessive stress can trigger the sympathetic adrenal medulla system and hypothalamus-pituitary adrenal axis, which cause physical and mental health problems (24). This interplay of physical and mental health leads to medical staff with poorer physical health to be more susceptible to mental health problems in response to stress compared to healthy medical staff. Secondly, we also found more anxiety and depression in male than female frontline medical staff, which is inconsistent with findings of other studies during the epidemic period (25–27). For example, Du et al. (25) surveyed 134 frontline medical workers during COVID-19 outbreak in Wuhan and found that anxiety and depression symptoms were more common among female medical staff than male medical staff, which is different from the present study. This discrepancy could be related to various assessment scales used, different samples selected and different data analyses used in these studies. In addition, due to convenient sampling in this study, a relatively small sample size of male frontline medical staff might lead to cases of bias. Thirdly, we found that married medical staff reported more mental health symptoms than those who were unmarried or divorced. This finding indicates perhaps that greater family responsibilities amplifies the level of perceived stress of medical staff, which in turn results in worse mental health. This finding is consistent with the existing literature, which suggests health care workers living with children were more concerned about their own health and that of their families (23).

The third important finding of the study is that frontline medical staff faced higher occupational stress during COVID-19 outbreak than non-frontline staff, specifically in terms of work hours, work difficulty, and occupational hazards. Furthermore, occupational stress acted as a risk factor for mental health symptoms in medical staff. Specifically, occupational hazards contribute to mental health symptoms in frontline medical staff but not in non-frontline medical staff. This result is consistent with the findings in Wuhan, which reported that occupational hazard was identified as a significant risk factor of anxiety in frontline medical staff (28). It could suggest that the lack of sufficient information of COVID-19, the high propagation potential of asymptomatic carriers, and the depletion of personal protection equipment increased the psychological symptoms burden of frontline medical staff (29). Compared with the frontline medical staff, the risk of exposure to infection is much lower among the non-frontline medical staff. Thus, it may not be a significant contributor of poor mental health in non-frontline medical staff. Meanwhile, it is worth mentioning that work difficulty was significantly associated with the mental health symptoms of both frontline and non-frontline medical staff. A pandemic renders essential workers' tasks more complex and difficult to manage, which may require them to have more energy to accomplish their work responsibilities. Such responsibilities may result in medical staff burnout and ultimately lead to anxiety, depression and other adverse emotions. Frontline staff, for example, may feel psychologically burdened over the responsibility of medical failures that may directly lead to health deterioration or death of their patients.

In addition, the increase in work hour and work intensity leads to the poor mental health of medical staff (30). This result is consistent with a growing literature showing that working longer hours each day is associated with significantly greater stress-related symptoms of medical staff, such as headache, and gastrointestinal upset (5).

There are some limitations in our study. First, our study was based on cross sectional design, which does not permit determination of the cause-and-effect relation between occupational stress and mental health. To clarify the causality, we need longitudinal data or panel data for further research. Secondly, data were self-reported in nature and respondents might exaggerate or conceal mental health symptoms, which may be subject to reporting bias. Future studies should consider triangulating self-reports with clinical records, and health and social services records. Nevertheless, the findings in our study do provide valuable information for policy makers and mental health professionals regarding the psychological impact of an infectious disease outbreak and the potential crisis-preparedness factors to consider in future biological disasters.

Despite these limitations, we believe that there are at least two major advantages gained from our study. Firstly, we characterize the feature of frontline medical staff who are more susceptible to mental health problems during epidemic, which may prompt the authorities to establish more rigorous standard for the selection of frontline medical staff from volunteers. For instance, the higher percentage of single, and good perceived physical health medical staff may be taken into consideration. Secondly, our study shows a novel association between working difficulty and mental health symptoms of medical staff. This finding suggests that even medical staff, one group of higher educated population, may feel more stressful to manage the complex and difficult tasks. The hospital administration should take steps to optimize the division of labor, and frame hierarchical decision making strategy.

## Implication and Conclusion

The major empirical findings lead to three critical implications. First, based on the findings that medical staff experienced high level of anxiety, depression and insomnia during the COVID-19 epidemic, the Chinese government may attend more to the growing concern of mental health among them by establishing mental health assessment and efficient psychological interventions in hospitals. This may be of particular salience for male, married medical staff with poorer physical health as they may experience more anxiety and depression symptoms. Second, in consideration that there is a significant association between occupational stress and mental health symptoms among the medical staff during the COVID-19 epidemic, favorable social support and response strategies are essential for reducing occupational stressors provisionally as well as lowering risk of long-lasting effects. The response to ongoing high stress should aim to support coping, foster resilience, reduce burnout and reduce the risk of developing mental health difficulties. Third, crisis-preparedness training is also essential to improve the mental health of the medical staff during a biological disaster. Crisis-preparedness training program not only includes the clinical skills required to deal with health crisis, but also the skills required to deal with the potentially traumatic situations that medical staff might be exposed to. In addition, this program would develop skills to cope with these and awareness of potential mental health consequences (31).

In conclusion, this study provides empirical evidence for the prevalence and severity of medical staff during the COVID-19 period. The frontline medical staff reported higher level of depression, anxiety and insomnia than the non-frontline medical staff and citizens during the COVID-19 epidemic. Furthermore, male, married medical staff with poorer physical health exhibited lower mental health. Four indicators on occupational stress acted as risk factors for mental health symptoms in medical staff.

## Data Availability Statement

The raw data supporting the conclusions of this article will be made available by the authors, without undue reservation.

## Ethics Statement

The study was approved by the Ethics Committee of the Wenzhou Medical University. The patients/participants provided their written informed consent to participate in this study.

## Author Contributions

LC, XZ, KZ, GZ, YL, WW, and RF: participated in research design. LC, WW, GZ, RF, DX, XL, and HH: collected the data. LC, YL, WW, XZ, KZ, GZ, and DX: conducted the data analysis. LC, YL, WW, XZ, KZ, GZ, and AN-A: wrote and contributed to the writing of the manuscript. All authors contributed to the article and approved the submitted version.

## Conflict of Interest

The authors declare that the research was conducted in the absence of any commercial or financial relationships that could be construed as a potential conflict of interest.
